# GOLink: Finding Cooccurring Terms across Gene Ontology Namespaces

**DOI:** 10.1155/2013/594528

**Published:** 2013-12-31

**Authors:** Richard W. Francis

**Affiliations:** Division of Bioinformatics and Biostatistics, Telethon Institute for Child Health Research, Centre for Child Health Research, The University of Western Australia, Perth 6008, Australia

## Abstract

The Gene Ontology (GO) provides a resource for consistent annotation of genes and gene products that is extensively used by numerous large public repositories. The GO is constructed of three subontologies describing the cellular component of action, molecular function, and overall biological process of a gene or gene product. Querying across the subontologies is problematic and no standard method exists to, for example, find all molecular functions occurring in a particular cellular component. GOLink addresses this problem by finding terms from all subontologies cooccurring with a term of interest in annotation across the entire GO database. Genes annotated with this term are exported and their GO annotation is assigned to three separate GOLink terms lists based on specific criteria. The software was used to predict the most likely Biological Process for a group of genes using just their Molecular Function terms giving sensitivity, specificity, and accuracy between 80 and 90% across all the terms lists. GOLink is made freely available for noncommercial use and can be downloaded from the project website.

## 1. Introduction

With the number of sequenced genomes at various stages of completion being in the tens of thousands [1] and the number of genomic features (genes, RNAs, etc.) identified across these genomes in the millions, the need for accurate and consistent genomic annotation is paramount.

The Gene Ontology (GO) [2] was created in 1998 by researchers at FlyBase, The Saccharomyces Genome Database (SGD), and The Mouse Genome Database as a collaborative effort to address the need for consistent descriptions of gene products across different databases. This group has since grown to include 26 consortium members and associates [3] and the GO is a key member of the Open Biological and Biomedical Ontologies (OBO) community [4].

In this context, an ontology can be defined as the specifications of a relational vocabulary [5]. Ontologies provide a controlled vocabulary for representing and communicating knowledge about a topic and a set of relationships that hold among the terms of the vocabulary. The topic for the GO is genes and gene products such as transcripts, proteins, or RNAs that are described in three related subontologies (also called namespaces), Biological Process, the broad biological system in which a gene product is involved; Molecular Function, the specific role a gene product has or potentially has within a Biological Process; and Cellular Component, the location in a cell where the gene product performs its Molecular Function. Each ontology is composed of nodes (terms) and edges (relationships) and is structured as a directed acyclic graph (DAG). As one moves down the nodes of the graph from a parent to a child, terms become increasingly more specific. A DAG allows for child terms to have more than one parent and this enables complex relationships to exist between them. The GO currently contains approximately 38 thousand terms that have been used by consortium members to annotate almost 25 million gene products [6]. Each annotation is accompanied by an evidence code to denote the method by which the annotation was made. Computationally sourced annotations, such as the evidence code IEA (inferred from electronic annotation), are regarded as less confident annotations than those that are assigned on the strength of experimental evidence. All data is publicly available for download and the GO team provides a number of entry points to query the data including an application programming interface (API) to permit custom queries to either a GO database or an ontology flat file [7].

The GO has been used in a number of analytical contexts [8]. The results of gene expression microarrays can be enriched with terms pertaining to Biological Process, which helps to discover if entire pathways are upregulated as opposed to simply individual genes. Subcellular location can be predicted for an unknown gene sequence by performing a similarity search such as BLAST and inferring from the Cellular Component GO terms of top hits.

There are some limitations to the GO, however. One such limitation is that, by design, there is no link between the three subontologies. This means one cannot directly answer questions such as “what are all the biological processes occurring in the cellular component ‘nucleus'?” Some attempts have been made to achieve this. Mungall [9] exploited the high degree of regularity in phrase structure of OBO term definitions and converted tokenized definitions from the GO, biochemical ontology, and the cell ontology into a language (Obol) that can be parsed computationally with a reasoner. This was used, amongst other things, to find missing relationships within the GO and between ontologies. Similarly, Wroe et al. [10] converted the GO to a DAML + OIL framework to enable reasoners to parse the ontology. Bada and Hunter [11] used regular expressions to find patterns in GO definitions and create an assertional model to integrate the three GO subontologies, the Chemical Entities of Biological Interest Ontology (ChEBI), and the Cell Type Ontology (CTO). Bada et al. [12] interrogated the GO, compiling groups of terms cooccurring in annotations of gene products, in order to suggest potential biologically relevant terms to annotators. Binns et al. [13] and Huntley et al. [14] describe functionality within the QuickGO tool from the GO Annotation (GOA) group [15] that provides information on how many times a query term cooccurs with other GO terms specifically across the UniProt Knowledgebase (UniProtKB) database [16]. This allows researchers to input a query term such as “nucleus” and view all terms that are frequently annotated alongside this term. However, there are limitations to this method, such as not being able to explore custom subsets of data within the UniProtKB database and a hard limit on the number of terms returned to the user.

This paper presents GOLink, an alternative tool for finding GO terms from across the three GO namespaces that cooccur with a given query term. GOLink differs from existing tools by using the GO API to mine the full complement of the GO database, using methods that take into account the namespace of a query term when assessing cooccurrence. The key advantages of using this particular method and source data are described along with other practical uses for the tool. These include predicting the most likely Biological Process for genes by using just their assigned Molecular Function terms, where GOLink achieves levels of specificity, sensitivity, and accuracy above 80%.

## 2. Methods

### 2.1. System Requirements

GOLink is written in Perl and requires the GO database API (go-db-perl) module (GO::AppHandle), available from the Comprehensive Perl Archive Network (CPAN).

GOLink requires access to a GO database (ideally locally installed). For the analyses in this paper, the GO database v201212 was downloaded from http://www.geneontology.org/GO.downloads.database.shtml and installed using the instructions at http://archive.geneontology.org/latest-full/README.

All analyses were run under 64-bit linux on a single 2.3 GHz core of a multicore AMD server; however, GOLink can be run on both Windows and MacOS platforms provided Perl, the required CPAN modules, and a GO database are installed.

GOLink is made freely available from the project website (http://bioinformatics.childhealthresearch.org.au/software/golink/) [17] under the GNU General Public License (GPL). All usage instructions are detailed in the software manual also available from the project website [17].

### 2.2. GOLink Analytical Method

**Figure 1:**
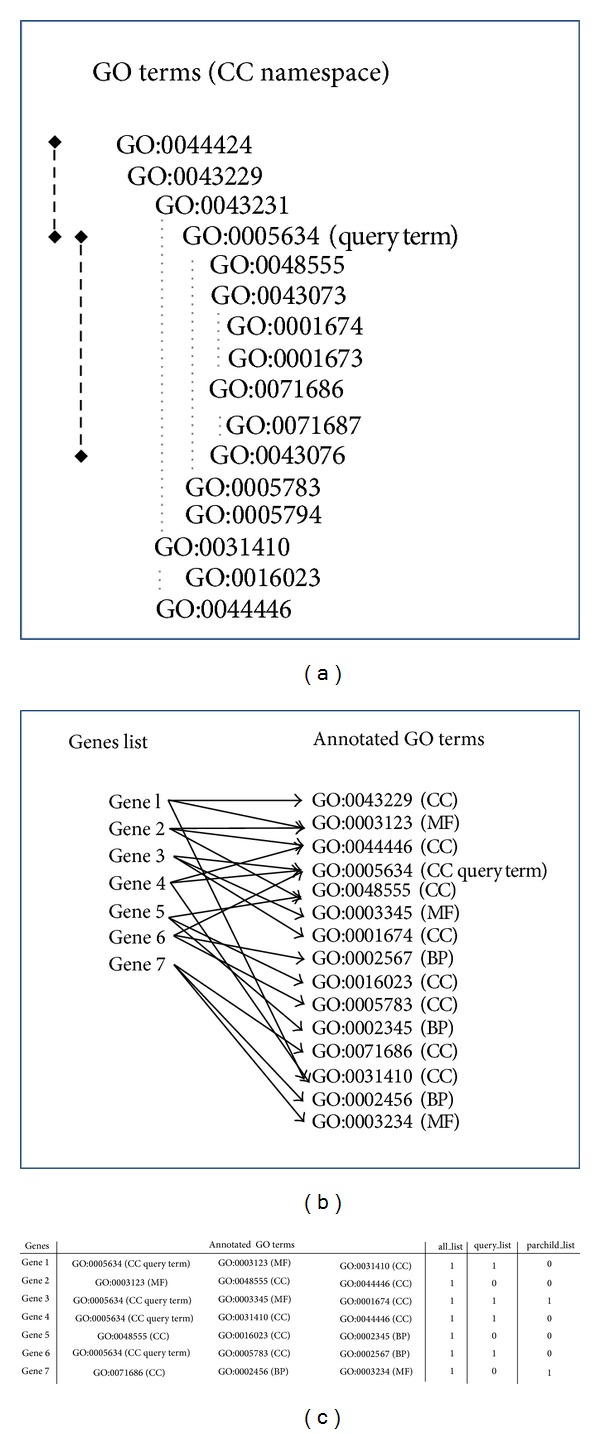
Summary of the GOLink stepwise protocol. (a) Simplified tree of a section of the Cellular Component (CC) GO DAG. The child terms of a supplied “query term” GO:0005634 (nucleus) are shown by the long black dashed line and its parents by the short black dashed line. (b) The GO terms used to annotate all genes annotated with the query term or any of its child terms are retrieved. Shown are the relationships between 7 genes and their corresponding GO annotation. The namespace to which a term belongs is indicated by the braced abbreviations Cellular Component (CC), Molecular Function (MF), and Biological Process (BP). (c) Summary of GO term annotation for each gene and an indication of which GOLink terms list each term will be contained in (see main text for classification rules).

The GOLink method is outlined in Figure 1. A user provides GOLink with a GO term such as GO:0005634 representing “nucleus,” which is referred to as the “query” term, along with any desired filters (evidence codes or annotation source databases—see below). GOLink will then retrieve all genes that have been annotated with the query term or any of its child terms (Figure 1(a)). Three separate GOLink “terms lists” are then created. Each gene is assessed in turn (Figure 1(b)) and its full complement of GO annotation is assigned to one or more terms list (Figure 1(c)) based on whether or not the annotation fulfills the list's specific criteria as follows.

1. all_list: stores all the gene's annotated GO terms.
2. query_list: stores all the gene's annotated GO terms only if the query term is one of those terms. This method is similar to QuickGO.
3. parchild_list: stores all the gene's annotated GO terms only if all terms within the same namespace as the query term are either one of the query term's parents or children.

In an iterative process, each list is gradually formed of “cooccurring” terms from all namespaces that fit the criterion above and that can subsequently be confidently associated with the initial query term. Once the lists are compiled, scores are calculated for terms in each list to allow them to be ranked. Calculations are based on those used for QuickGO's own cooccurring terms lists.

PR—empirical probability ratio:

(1) $$\begin{array}{c} \frac{\left(T/Q\right)}{\left(C/A\right)}. \end{array}$$

*S*%—empirical probability similarity ratio:

(2) $$\begin{array}{c} \left(\frac{T}{\left(Q+C-T\right)}\right)*100, \end{array}$$

where, given term *X* in the context of a single list, *Q* = the number of gene products fulfilling the criteria of the list; *T* = the number of gene products fulfilling the criteria of the list that contain term *X*; *C* = the number of gene products annotated with term *X* in the entire GO database, given any filters; and *A* = the number of gene products in the entire GO database, given any filters.

The Empirical Probability Ratio is a measure of how similar *T* is with *C*. While being a useful score this penalises interesting candidates that have high *C* but a slightly lower value of *T*. This discrepancy is addressed in the Empirical Probability Similarity Ratio, which not only accounts for the relationship between *T* and *C* but gives higher scores where *T* and *Q* are also similar in value.

### 2.3. GOLink Filters

Users can explore subsets of GO annotation data by optionally applying a choice of three filters. The namespace filter controls the nature of the terms output to the final terms list. Unfiltered, GOLink will return terms from all namespaces that cooccur with the query term. However, users can request that only terms from a limited set of the three namespaces are returned. When a GO term is assigned to a gene product by a GO consortium member (e.g., FlyBase, SGD) an evidence code is also attributed. GOLink provides a database filter to target annotations made by a particular consortium member and an evidence code filter to allow fine-grained control on the level of quality of terms returned. For convenience a “!” can be specified to not include terms from a particular database or evidence code. For example, when applying the evidence code filter “!IEA” GOLink will only consider terms that are not attributed with the evidence code IEA (inferred from electronic annotation). Given that the IEA evidence code suggests a less confident term assignment, using “!IEA” is recommended for any analyses with GOLink.

### 2.4. Data Interpretation

GOLink terms lists can be ordered as required; however, it is recommended that results are sorted by descending Empirical Probability Similarity Ratio (*S*%) and then descending Empirical Probability Ratio (PR). This serves to place the terms most strongly linked to the query term at the top of the list and also mirrors the ordering method used by QuickGO. GOLink outputs three terms lists, each with related yet contrasting results due to the differing criteria used to compile each list. When reviewing these lists it is important to take the initial query term used for the analysis into account. If the query term originates from towards the top of GO DAG, it will be more likely to have a large number of child terms, which inevitably results in many more terms being returned to all the GOLink terms lists than for more specific query terms at lower levels of the GO graph. In this case using the more stringent parchild_list may be preferable. Conversely, lower level terms are more likely to not return any results to the more stringent GOLink terms lists due to there being fewer annotations where specific criteria are met. Here the all_list will contain the most comprehensive data.

### 2.5. GOLink Terms List Comparisons

GOLink terms lists were generated using the Biological Process query term “regulation of gene expression” (GO:0010468) with the evidence code filter “!IEA” and database filter “UniProtKB” being applied. Comparisons of GOLink terms lists were performed using a three-way Venn diagram of all terms in each list using Venny [18].

### 2.6. Statistical Testing

A Perl script was created that, given an initial GO term, pulls all genes annotated with that term from the GO database along with their other annotated GO terms. Using this script a “positive” and a “negative” list of genes and their GO annotation were generated using the initial Biological Process terms “regulation of gene expression” (GO:0010468) for the positive list and a combination of “lipid metabolic process” (GO:0006629) and “cell motility” (GO:0048870) for the negative list. Genes were only included in each list if they had at least one Molecular Function term as part of their annotation. GOLink terms lists were then generated using the Biological Process query term “regulation of gene expression” (GO:0010468), opting to only output Molecular Function terms. In all cases the evidence code filter “!IEA” and database filter “UniProtKB” were applied and output terms sorted as recommended above.

A gene in the positive or negative list was deemed as having a role in the “regulation of gene expression” if any of its Molecular Function terms matched either the first 10, 20, 30, 40, or 50 (separately) Molecular Function terms in each of the three GOLink terms lists. Those genes in the negative list providing positive results were manually checked in UniProtKB [16] to ensure that they did not also have a role in the “regulation of gene expression” as well as either “lipid metabolic process” or “cell motility.”

A statistical assessment of performance was calculated as follows:

(3) $$\begin{array}{c} \text{Sensitivity}=\frac{\text{TP}}{\left(\text{TP}+\text{FN}\right)},\\ \text{Specificity}=\frac{\text{TN}}{\left(\text{FP}+\text{TN}\right)},\\ \text{Positive}\;\text{Predictive}\;\text{Value}\;\left(\text{PPV}\right)=\frac{\text{TP}}{\left(\text{TP}+\text{FP}\right)},\\ \text{Negative}\;\text{Predictive}\;\text{Value}\;\left(\text{NPV}\right)=\frac{\text{TN}}{\left(\text{TN}+\text{FN}\right)},\\ \text{Accuracy}=\frac{\left(\text{TP}+\text{TN}\right)}{\left(\text{TP}+\text{TN}+\text{FP}+\text{FN}\right)}, \end{array}$$

where TP = number of genes correctly predicted to be involved with the regulation of gene expression; TN = Number of genes correctly predicted to be not involved with the regulation of gene expression; FP = number of genes incorrectly predicted to be involved with the regulation of gene expression; and FN = number of genes incorrectly predicted to not be involved with the regulation of gene expression.

In this context, sensitivity refers to how likely GOLink predicts a role in the regulation of gene expression for those genes in the positive list (TP), specificity measures how well GOLink predicts no role in the regulation of gene expression for genes in the negative list (TN), PPV assesses the chance that a gene is involved with regulation of gene expression given a prediction that it is involved, and NPV calculates the chance that a gene is not involved with regulation of gene expression given a prediction that it is not involved in that process. Mixed bar and line graphs displaying the results of this assessment were produced using *R* [19].

### 2.7. Exploring Source Database Filters

For the annotation source databases “SGD” (Saccharomyces Genome Database [20]), “PomBase” (main resource for the fission yeast *Schizosaccharomyces pombe* [21]), “MGI” (Mouse Genome Informatics [22]), “ZFIN” (The Zebrafish Model Organism Database [23]), and “UniProtKB” [16], separate GOLink terms lists were generated using the Biological Process query term “regulation of gene expression” (GO:0010468) and applying the evidence code filter “!IEA.” Using data from each respective all_list, pairwise comparisons were made between terms from UniprotKB and each of the other source databases using Venny [18].

### 2.8. Comparison to QuickGO

A QuickGO (released on Friday, 04 January 2013) terms list was exported by using the query term “regulation of gene expression” and selecting from the “!IEA” list. An equivalent GOLink dataset was generated by using the query term “regulation of gene expression” and applying the “!IEA” evidence code filter with results being sorted as recommended above. The position at which each of the 100 QuickGO terms fell in the sorted GOLink list was then obtained. The GOLink “query_list” terms list was used to compare against the QuickGO terms list as the term assignment criteria for both lists are the most comparable.

The exported QuickGO terms list was also subjected to a separate similar statistical assessment to that described above, whereby a gene in the positive or negative list was deemed as having a role in the “regulation of gene expression” if any of its Molecular Function terms matched any of the Molecular Function terms in the QuickGO terms list.

## 3. Results

### 3.1. GOLink Term List Comparisons

**Figure 2:**
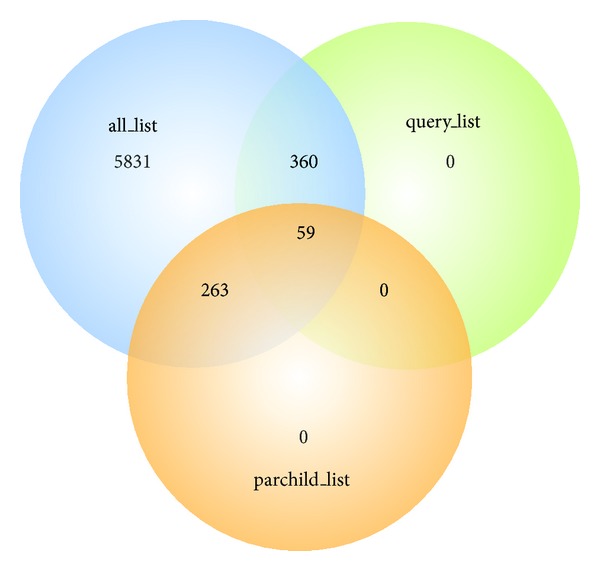
Venn diagram displaying overlapping terms across the three GOLink terms lists generated using the query term “regulation of gene expression” (GO:0010468) and applying the evidence code filter “!IEA” and database filter “UniProtKB.” The all_list, query_list, and parchild_list returned 6513, 419, and 322 terms, respectively.

Tables 1(a)–1(c) show the top 10 GOLink cooccurring terms and their scores in each terms list generated using the query term “regulation of gene expression” (GO:0010468) and applying the evidence code filter “!IEA” and database filter “UniProtKB.” The complete lists can be found in Supplementary File 1 (see Supplementary Material available online at http://dx.doi.org/10.1155/2013/594528). Overall the all_list, query_list and parchild_list, returned 6513, 419, and 322 terms, respectively.  Figure 2 shows a Venn diagram comparing the generated lists. As expected there are a large number of unique terms in the all_list given the comparatively large number of terms returned and there are no unique terms found within either the query_list or parchild_list. The 360 terms overlapping the all_list and query_list in the Venn diagram (Figure 2) represent terms from gene products that are annotated with the query term but have other terms in the Biological Process namespace that are not either a parent or a child of the query. The 263 terms (Figure 2) overlapping the all_list and parchild_list represent terms from gene products that were not directly annotated with the query term but with one of its child terms. Overall, there were 59 consensus terms across the three lists, which are listed in Supplementary Table 1.

### 3.2. Statistical Testing

A useful application of the GOLink term lists is, for example, to use a gene's existing Molecular Function GO terms to establish whether it has a role in a particular Biological Process, such as the “regulation of gene expression.” If a gene has been annotated with only Molecular Function terms, one can compare these terms to a GOLink terms list for a Biological Process query term of interest. If the Molecular Function terms are found in the GOLink terms list, one can infer that the gene's function may be associated with the initial Biological Process query term.

**Figure 3:**
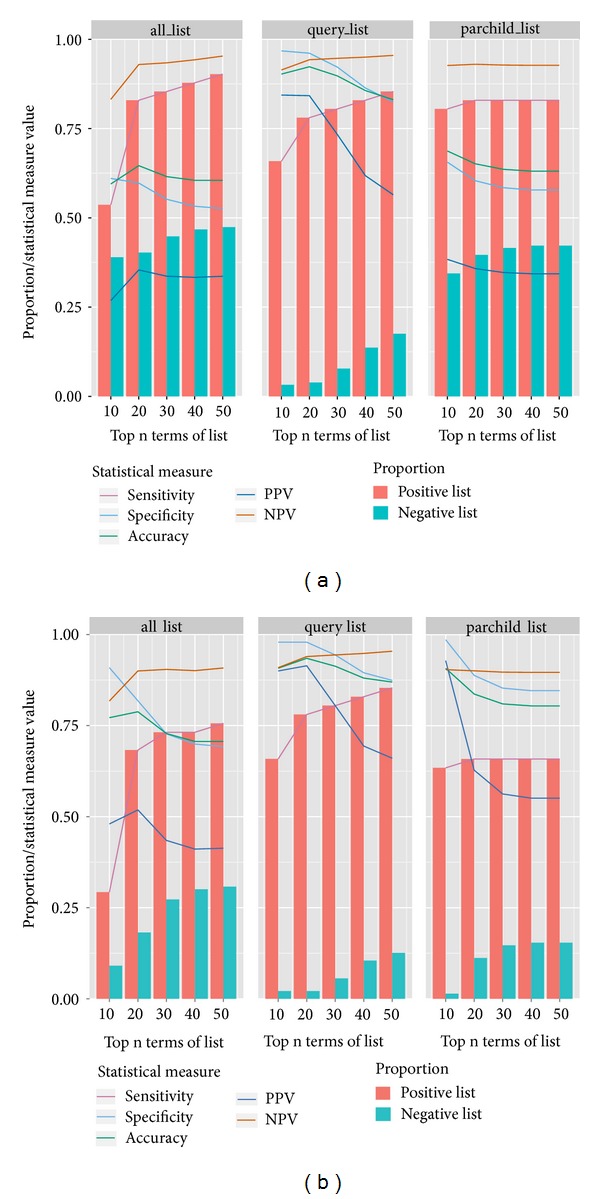
Statistical assessment of GOLink's ability to predict whether or not a gene has a role in a particular Biological Process based on its Molecular Function terms. GO annotation terms from genes in a positive and a negative list were matched against terms in the three GOLink terms lists. (a) The measures of sensitivity, specificity, accuracy, positive predictive value, and negative predictive value as well as the proportion of genes reported in the positive and negative lists. (b) The same measures but showing the impact following the removal of 11 known true positives from the negative list and the removal from the GOLink terms lists of the term “protein binding,” which has relevance across both the positive and negative gene lists.

Figure 3(a) and Supplementary File 2 show the sensitivity, specificity, PPV, NPV, and accuracy of such an assessment matching only the Molecular Function terms from the three GOLink terms lists with a positive and negative list of genes and their annotated GO terms. They also show the proportion of genes found in each of the positive and negative lists by the three GOLink terms lists. The positive list contained 41 genes (1163 terms, 154 MF terms) annotated as having a role in the regulation of gene expression, while the negative list contained 154 individual genes (2723 terms, 503 Molecular Function terms). The genes contained in the positive and negative lists and their associated GO annotation can be found in Supplementary File 2. The top 50 Molecular Function terms from each of the three GOLink terms lists that were used in the matching process are also shown in Supplementary File 2 and a Venn diagram showing their overlap can be found in Supplementary Figure 1. It should be noted that the rationale behind using only up to the first 50 Molecular Function terms from the GOLink terms lists in the matching process was simply that each list contained at least 50 terms.

Overall the query_list outperformed the other two GOLink terms lists in predicting genes with a role in the regulation of gene expression using only their assigned Molecular Function terms. For this list, sensitivity, specificity and accuracy all averaged between ~80% and 90% with the highest values occurring with comparisons using the top 30 GOLink Molecular Function terms. Using these top 30 terms in the query_list yielded 33/41 true positives and only 12/154 false positives giving a PPV of 73% and a NPV of 94%. The all_list and parchild_list showed increasingly higher sensitivity as more terms were added in to the comparison, but this was at the expense of specificity, which remained above 50% but much lower than that of the query_list. Eleven of the 154 negative genes were deemed to be actually true positives (Supplementary Table 2) with a role in the regulation of gene expression as well as either “cell motility” or “lipid metabolic process” despite all but one not being directly annotated with “regulation of gene expression.” These genes (such as Sterol regulatory element-binding proteins 1 and 2 and COUP transcription factor 2) were removed from their respective negative lists and the statistics recalculated, which served to increase values marginally across the board (Table (a) in Supplementary File 3). Most of the false positive genes predicted by the GOLink terms lists were annotated with the GO term “protein binding” (GO:0005515). This fairly generic term has relevance for both the negative and positive lists so users of the software are cautioned to consider this when performing analyses of this nature and may opt to remove terms from the GOLink lists where appropriate. Indeed removing both the false positives from the negative list and removing the GO term “protein binding” from the GOLink terms lists have a substantial impact on the statistical assessment across the board as can be seen in Figure 3(b).

### 3.3. Exploring Source Database Filters

**Figure 4:**
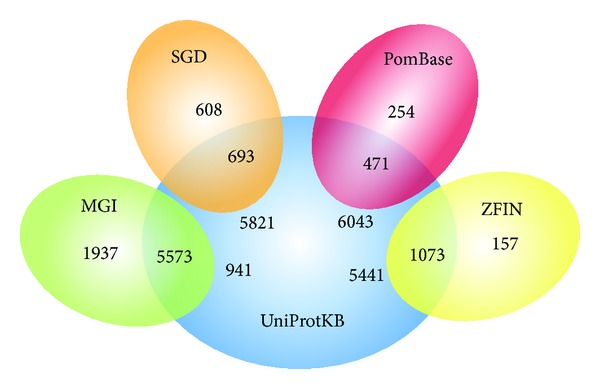
The effect of applying GOLink's annotation source database filter. Results from four annotation sources (“PomBase,” “MGI,” “SGD,” and “ZFIN”) are compared in separate pairwise Venn diagrams to results obtained using “UniProtKB” as the annotation source. All analyses used the query term “regulation of gene expression” and applied the “!IEA” evidence code filter. As expected, the majority of terms in the results of each compared source database could also be found in UniProtKB with unique terms having relevance to each respective annotation source.

Figure 4 shows the overlap of GOLink terms obtained using four species specific annotation source databases (MGI, PomBase, ZFIN, and SGD) in comparison with terms obtained from the more broad UniProtKB database, using the same query term (“regulation of gene expression”) and evidence code filter (“!IEA”). The full results for each analysis can be found in Supplementary File 4. For each of the species specific databases Figure 4 shows that, as expected, the majority of terms (between 53% and 87%) in their respective lists were also in the UniProtKB list. This group represents those terms that cooccur with the query term in annotation within both the species specific database and in annotation by UniProtKB for other species. The remaining terms are either unique to the species specific database or to UniprotKB. In the context of the species specific database these unique terms are either highly relevant to the species and only ever likely to have been used in annotation by the curators of the species specific database (e.g., “ascospore formation” within PomBase and SGD, “fin development” within ZFIN) or represent terms that only cooccur with the query term in this database compared to UniProtKB. Conversely, those terms unique to UniProtKB represent terms highly irrelevant to the species specific database (e.g., “viral reproduction” in all cases) or similarly only cooccur with the query term within UniProtKB assigned annotation.

### 3.4. Comparison to QuickGO

**Figure 5:**
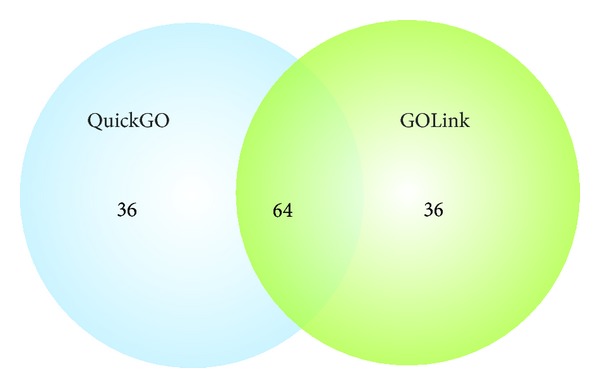
Venn diagram comparing the top 100 cooccurring terms returned by GOLink and QuickGO given similar input parameters. As expected there is a high level of overlap in the terms returned by both lists with discrepancies arising as a result of differences in the underlying databases used by the two tools.

Tables (a)–(c) in Supplementary File 5 show the full GOLink query_list and QuickGO terms lists used in the comparison ((b) and (c)) and a combined summary detailing the positions of the top 100 QuickGO terms as found in the GOLink query_list (a). Overall the vast majority (72%) of QuickGO terms are confined to the top 200 GOLink terms and Figure 5 shows that there is a 64% overlap between the top 100 QuickGO and GOLink terms. While all 100 QuickGO terms are represented somewhere in the GOLink query list, Table (a) in Supplementary File 5 shows that there are distinct differences in the ordering of the results within the more significant first 100 terms. This can be explained by the way protein identifiers are mapped within the database underlying QuickGO. The database used by GOLink is maintained by the GO consortium. Within this database, GO annotation is assigned to single gene product identifiers. The database for QuickGO is maintained by the GOA team and provides high quality annotation for all proteins within UniProt. Here, single protein identifiers are first mapped to UniProt accessions, and in many cases one identifier can map to several UniProt accessions (since there is likely to be several matches in TrEMBL, the nonreviewed section of UniProt). In these cases the same or similar annotation is assigned to each of the UniProt accessions. While appropriate in the context of the GOA project this leads to more redundancy in annotation in the database used by QuickGO than within that used by GOLink. Consequently, when considering cooccurring terms in QuickGO, terms from those genes that have multiple entries in TrEMBL will appear to cooccur more often than is the case within the GO database, which may not be desired. For example, in Table (a) in Supplementary File 5, QuickGO reports that the term “forebrain development,” which appears second in the QuickGO terms list, has been used to annotate 308 proteins within the GOA database. This term appears tenth in the GOLink list, where GOLink reports its assignment to 200 gene products within the GO database. Furthermore QuickGO and GOLink report that this term cooccurs in annotation with the query term on 56 and 26 occasions respectively. On closer examination, most of these gene products have GO terms assigned by MGI. Multiple UniProt accession numbers exist that map to single MGI identifiers annotated with the term “forebrain development” and this has the effect of inflating the number of cooccurrences with the query term, thus influencing the *S*% calculation and positioning it higher in the QuickGO terms list. A more extreme example can be seen for the term “spermatid differentiation” which appears at position 11 in the QuickGO list but position 531 in the GOLink list. In addition, the result of the PR calculation is altered as a consequence of the increased number of proteins within the GOA database. As both QuickGO and GOLink order their results by *S*% and then by PR and QuickGO only provides the first 100 hits, this explains why equivalent search parameters on these similar but different source databases lead to similar but differing results for GOLink compared to QuickGO.

In order to test QuickGO's ability to predict a gene's Biological Process from its Molecular Function a similar matching process to that undertaken for GOLink was performed with the top 100 cooccurring terms from QuickGO. Only 9 of the 41 positive genes were found using these terms, which consequently gave lower comparative performance scores to GOLink (data not shown), however it should be noted that as seen in Table (c) in Supplementary File 5 this list of terms only contained 4 Molecular Function terms to use in the comparison process.

## 4. Discussion

The Gene Ontology is a dynamic, growing resource for the annotation of genes and gene products. It is organized into three subontologies describing the cellular location of action of a gene or gene product, the main process it is involved in, and its role in this process. In order for the three subontologies to remain orthogonal, there are no designed links between them. However, this prevents legitimate attempts to answer queries involving data from more than one of the subontologies.

This paper discusses a new tool, GOLink, which uses the GO Perl API to explore the GO database and implement three increasingly stringent methods to compile lists of GO terms cooccurring in gene product annotations with a provided query term. When given an initial query term, the three methods store either all terms cooccurring with the query term or any of its child terms (all_list), only those terms cooccurring with query term (query_list) or only those terms cooccurring in annotations with the query term or its child terms where other annotated terms in the same namespace (Cellular Component, Molecular Function, and Biological Process) as the query term are limited to the query term and any of its parent or child terms (parchild_list).

The GOLink query_list works in a similar way to the method employed by QuickGO. However, the key difference between the remaining GOLink lists and QuickGO is that the latter uses a pool of terms from genes directly annotated with the query term, whereas the GOLink all_list and parchild_list use a pool derived from genes annotated with the query term and/or its child terms. This pool is then tested for cooccurring terms that fit GOLink list criteria. The main reason GOLink uses this extended pool is to not penalize the partonomic/taxonomic relationship between a term and its child terms. Furthermore, this allows GOLink to apply further logical stringency in the parchild_list to specifically exclude occurrences where the query term is annotated together with terms in the same namespace that are neither a parent nor a child of the query term. For example, if a gene performs a particular function and operates both in the cytoplasm and the nucleus it may well be annotated with 2 Cellular Component terms (nucleus and cytoplasm) and at least 1 Molecular Function term. In this case the Molecular Function term cannot be exclusively linked with either of the Cellular Component terms as the Molecular Function term may only be relevant for the gene's function in the cytoplasm. If this distinction is required then the parchild_list will take this into account, whereas this functionality is not possible using QuickGO.

The other main advantages of GOLink over QuickGO lie in the distinct differences between the underlying databases of the two software and the availability of flexible methods to filter the terms assessed and those returned. The process of identifier mapping in the GOA database that provides the annotation used by QuickGO leads to an increased redundancy of annotation across this database as single gene product identifiers commonly map to multiple UniProt accessions. This results in a potentially inflated cooccurrence of GO terms as demonstrated in this paper. Results provided by GOLink are not prone to this issue. QuickGO provides two results sets; one unfiltered list of cooccurring terms and one with a “!IEA” filter applied to discount associations from purely computationally assigned terms. For both, only the first 100 cooccurring terms are returned. GOLink is much more flexible in that not only does it produce multiple terms lists of varying stringency, but it also provides fine-grained customizable filtering. The database filter allows a user to identify cooccurring terms within the context of a particular species that could be diluted if searching within a broader context such as the full UniProt database. The evidence code filter gives precise control over the quality of annotation considered during an analysis using the wide variety of evidence codes provided within the GO database. Both of these filters are not available in QuickGO. Finally, applying the GOLink namespace filter will only return results from a defined set of the three available namespaces. QuickGO only returns the first 100 cooccurring terms from all namespaces. If an analysis only called for examining cooccurring Molecular Function terms then this assessment can only be confidently made using GOLink as the full complement of cooccurring Molecular Function terms may not be available within the static top 100 results gleaned from QuickGO. This limitation of QuickGO also hinders its use in the types of analyses undertaken in the statistical assessment of GOLink to predict, for example, a gene's Biological Process from its assigned Molecular Function terms, as there may not be sufficient Molecular Function terms returned. QuickGO cooccurring terms are immediately available via the QuickGO web interface, whereas the GOLink algorithm can take a number of hours to run for some analyses. There are a number of sections of the GOLink algorithm; however, that lend themselves to parallel processing. This will be available in future versions of the software; however, its absence in the current version does not hinder the tool's utility.

The GOLink method performs very well in predicting the Biological Process from a gene's assigned Molecular Function terms, showing both high sensitivity and specificity. This application of GOLink is equivalent to first predicting Molecular Function terms for an unknown gene using, for example, InterProScan [24] then using GOLink to generate terms lists for a Biological Process query term of interest and then predicting the likelihood of the unknown gene being involved in that particular Biological Process. Similarly using a Cellular Component query term and comparing the GOLink terms with InterProScan or otherwise derived GO terms, a researcher can predict the Cellular Component of a gene. In cases where a more direct prediction of, for example, Biological Process is required, multiple Molecular Function query terms could be provided to GOLink with the most likely associated Biological Process being the highest scoring consensus term found in all terms lists. In addition, as Cellular Component, Molecular Function, and Biological Process terms can be incorporated into the GOLink terms lists, in a similar method to Bada et al. [12], GOLink can be used to help annotators choose potential terms to assign to a gene. Furthermore, it was discovered that a number of false positives found in the statistical analysis were not specifically assigned the term “regulation of gene expression” but clearly have a role in this process. Therefore, GOLink can be used to correct/amend annotations of gene products.

## 5. Conclusion

GOLink is a Perl based tool that finds terms cooccurring with a given query term in annotation across the full complement of the Gene Ontology database. It has advantages over other existing tools and can be used in a variety of applications. GOLink is open source and freely available from the project website.

## Supplementary Material

Supplementary File 1 (SupplementaryFile1.xls): GOLink terms lists generated using the query term ‘regulation of gene expression', the evidence code filter “!IEA” and database filter ‘UniProtKB'.
Supplementary File 2 (SupplementaryFile2.xls): The top 50 Molecular Function terms from each GOLink terms list (a); the genes and annotation used for the positive list and the negative list (b); the raw statistical data for sensitivity, specificity, accuracy, PPV, NPV and the number and proportion of positive and negative genes found (c). These latter data correspond to Figure 3.
Supplementary File 3 (SupplementaryFile3.xls): The genes and annotation used for the positive list and the negative list and raw statistical data for sensitivity, specificity, accuracy, PPV, NPV and the number and proportion of positive and negative genes found when the true positives from Supplementary Table 2 are removed (a). The raw statistical data when both the true positives and the term ‘protein binding' are removed from the GOLink terms lists (b).
Supplementary File 4 (SupplementaryFile4.xls): The five GOLink all_list terms lists generated using the query term ‘regulation of gene expression' and the evidence code filter “!IEA” for each of the five annotation sources (“UniprotKB, “PomBase”, “MGI”, “SGD” and “ZFIN”) used in the creation of Figure 4.
Supplementary File 5 (SupplementaryFile5.xls): a) Summary of the top 100 terms from a GOLink query_list terms list (b) and a QuickGO terms list (c) generated using the query term ‘regulation of gene expression' and the evidence code filter “!IEA”.
Supplementary Figure 1 (SupplementaryFigure1.jpg): Venn diagram showing the overlap between the top 50 Molecular Function terms from each of the three GOLink terms lists as found in Supplementary File 2.
Supplementary Table 1 (SupplementaryTable1.doc): The 59 GOLink consensus terms found in all three GOLink terms lists.
Supplementary Table 2 (SupplementaryTable2.doc): Genes from the negative list deemed to be true positives following manual assessment in UniProtKB.

## Figures and Tables

**Table 1a:** (a)

Namespace	GO_ID	GO_Name	Score (PR)	Score (*S*%)
cellular_component	GO:0005634	Nucleus	5.19	22.32
biological_process	GO:0006355	Regulation of transcription, DNA-dependent	16.53	20.96
biological_process	GO:0045893	Positive regulation of transcription, DNA-dependent	16.53	20.7
molecular_function	GO:0003700	Sequence-specific DNA binding transcription factor activity	14.98	20.31
biological_process	GO:0045944	Positive regulation of transcription from RNA polymerase II promoter	16.53	18.12
biological_process	GO:0045892	Negative regulation of transcription, DNA-dependent	16.53	17.17
cellular_component	GO:0005737	Cytoplasm	3.29	12.55
molecular_function	GO:0005515	Protein binding	3.14	12.49
biological_process	GO:0000122	Negative regulation of transcription from RNA polymerase II promoter	16.53	12.06
molecular_function	GO:0003677	DNA binding	10.00	10.11

**Table 1b:** (b)

Namespace	GO_ID	GO_Name	Score (PR)	Score (*S*%)
biological_process	GO:0010468	Regulation of gene expression	1556.26	100
biological_process	GO:0060059	Embryonic retina morphogenesis in camera-type eye	1141.26	19.3
biological_process	GO:2000744	Positive regulation of anterior head development	1556.26	18.87
biological_process	GO:0072049	Comma-shaped body morphogenesis	1556.26	18.87
biological_process	GO:0072050	S-shaped body morphogenesis	1556.26	18.87
biological_process	GO:0021937	Cerebellar Purkinje cell-granule cell precursor cell signaling involved in regulation of granule cell precursor cell proliferation	1556.26	18.87
biological_process	GO:0072077	Renal vesicle morphogenesis	1556.26	18.87
biological_process	GO:0021527	Spinal cord association neuron differentiation	1414.79	18.52
biological_process	GO:0061205	Paramesonephric duct development	1296.89	18.18
biological_process	GO:0001705	Ectoderm formation	1296.89	18.18

**Table 1c:** (c)

Namespace	GO_ID	GO_Name	Score (PR)	Score (*S*%)
biological_process	GO:0006355	Regulation of transcription, DNA-dependent	34.60	28.54
molecular_function	GO:0003700	Sequence-specific DNA binding transcription factor activity	27.11	22.2
biological_process	GO:0045893	Positive regulation of transcription, DNA-dependent	8.57	5.78
biological_process	GO:0045892	Negative regulation of transcription, DNA-dependent	9.44	5.76
biological_process	GO:0006446	Regulation of translational initiation	38.20	5.47
molecular_function	GO:0003677	DNA binding	8.62	5.36
molecular_function	GO:0003743	Translation initiation factor activity	25.07	4.72
cellular_component	GO:0005852	Eukaryotic translation initiation factor 3 complex	33.37	4.57
biological_process	GO:0000122	Negative regulation of transcription from RNA polymerase II promoter	8.19	3.98
biological_process	GO:0006357	Regulation of transcription from RNA polymerase II promoter	12.52	3.94
