# Supplementary material for: GOLink: Finding Cooccurring Terms across Gene Ontology Namespaces
Source: Int J Genomics. 2013 Dec 31;2013:594528. doi: 10.1155/2013/594528 (PMC3892482; doi:10.1155/2013/594528)
Supplement: Supplementary file 1 — (SupplementaryFile1.xls): GOLink terms lists generated using the query term ‘regulation of gene expression', the evidence code filter “!IEA” and database filter ‘UniProtKB'. Supplementary File 2 (SupplementaryFile2.xls): The top 50 Molecular Function terms from each GOLink terms list (a); the genes and annotation used for the positive list and the negative list (b); the raw statistical data for sensitivity, specificity, accuracy, PPV, NPV and the number and proportion of positive and negative genes found (c). These latter data correspond to Figure 3. Supplementary File 3 (SupplementaryFile3.xls): The genes and annotation used for the positive list and the negative list and raw statistical data for sensitivity, specificity, accuracy, PPV, NPV and the number and proportion of positive and negative genes found when the true positives from Supplementary Table 2 are removed (a). The raw statistical data when both the true positives and the term ‘protein binding' are removed from the GOLink terms lists (b). Supplementary File 4 (SupplementaryFile4.xls): The five GOLink all_list terms lists generated using the query term ‘regulation of gene expression' and the evidence code filter “!IEA” for each of the five annotation sources (“UniprotKB, “PomBase”, “MGI”, “SGD” and “ZFIN”) used in the creation of Figure 4. Supplementary File 5 (SupplementaryFile5.xls): a) Summary of the top 100 terms from a GOLink query_list terms list (b) and a QuickGO terms list (c) generated using the query term ‘regulation of gene expression' and the evidence code filter “!IEA”. Supplementary Figure 1 (SupplementaryFigure1.jpg): Venn diagram showing the overlap between the top 50 Molecular Function terms from each of the three GOLink terms lists as found in Supplementary File 2. Supplementary Table 1 (SupplementaryTable1.doc): The 59 GOLink consensus terms found in all three GOLink terms lists. Supplementary Table 2 (SupplementaryTable2.doc): Genes from the negative list deemed t [file 594528.f1.zip › Tables/SupplementaryTable1.pdf]

Supplementary Table 1: The 59 common terms found in all three GOLink terms lists (all_list, query_list and parchild_list) using the query term ‘regulation of gene expression’ (GO:0010468) and applying the evidence code filter “!IEA” and database filter “UniProtKB”:

Namespace	GO_ID	GO_Name
**cellular_component**	GO:0005634	nucleus
**biological_process**	GO:0006355	regulation of transcription, DNA-dependent
**biological_process**	GO:0045893	positive regulation of transcription, DNA-dependent
**molecular_function**	GO:0003700	sequence-specific DNA binding transcription factor activity
**biological_process**	GO:0045944	positive regulation of transcription from RNA polymerase II promoter
**biological_process**	GO:0045892	negative regulation of transcription, DNA-dependent
**cellular_component**	GO:0005737	cytoplasm
**molecular_function**	GO:0005515	protein binding
**biological_process**	GO:0000122	negative regulation of transcription from RNA polymerase II promoter
**molecular_function**	GO:0003677	DNA binding
**cellular_component**	GO:0005654	nucleoplasm
**cellular_component**	GO:0005829	cytosol
**biological_process**	GO:0006357	regulation of transcription from RNA polymerase II promoter
**cellular_component**	GO:0005730	nucleolus
**molecular_function**	GO:0008134	transcription factor binding
**molecular_function**	GO:0043565	sequence-specific DNA binding
**cellular_component**	GO:0005667	transcription factor complex
**molecular_function**	GO:0003713	transcription coactivator activity
**molecular_function**	GO:0044212	transcription regulatory region DNA binding
**cellular_component**	GO:0005615	extracellular space
**biological_process**	GO:0010628	positive regulation of gene expression
**cellular_component**	GO:0005886	plasma membrane
**molecular_function**	GO:0042803	protein homodimerization activity
**molecular_function**	GO:0003705	RNA polymerase II distal enhancer sequence-specific DNA binding transcription factor activity
**molecular_function**	GO:0003714	transcription corepressor activity
**cellular_component**	GO:0048471	perinuclear region of cytoplasm
**molecular_function**	GO:0042802	identical protein binding

**molecular_function**	GO:0008270	zinc ion binding
**molecular_function**	GO:0046982	protein heterodimerization activity
**cellular_component**	GO:0043234	protein complex
**cellular_component**	GO:0005576	extracellular region
**biological_process**	GO:0051091	positive regulation of sequence-specific DNA binding transcription factor activity
**cellular_component**	GO:0005794	Golgi apparatus
**biological_process**	GO:0010629	negative regulation of gene expression
**biological_process**	GO:0043433	negative regulation of sequence-specific DNA binding transcription factor activity
**cellular_component**	GO:0005887	integral to plasma membrane
**molecular_function**	GO:0042393	histone binding
**biological_process**	GO:0010468	regulation of gene expression
**molecular_function**	GO:0008201	heparin binding
**molecular_function**	GO:0008022	protein C-terminus binding
**molecular_function**	GO:0004879	ligand-activated sequence-specific DNA binding RNA polymerase II transcription factor activity
**molecular_function**	GO:0001071	nucleic acid binding transcription factor activity
**cellular_component**	GO:0016021	integral to membrane
**molecular_function**	GO:0046983	protein dimerization activity
**molecular_function**	GO:0047485	protein N-terminus binding
**cellular_component**	GO:0000785	chromatin
**molecular_function**	GO:0035035	histone acetyltransferase binding
**biological_process**	GO:0061418	regulation of transcription from RNA polymerase II promoter in response to hypoxia
**biological_process**	GO:0000083	regulation of transcription involved in G1/S phase of mitotic cell cycle
**cellular_component**	GO:0005635	nuclear envelope
**cellular_component**	GO:0005618	cell wall
**molecular_function**	GO:0035198	miRNA binding
**molecular_function**	GO:0035257	nuclear hormone receptor binding
**cellular_component**	GO:0031519	PcG protein complex
**molecular_function**	GO:0020037	heme binding
**cellular_component**	GO:0035102	PRC1 complex
**cellular_component**	GO:0044428	nuclear part

**cellular_component**	GO:0015030	Cajal body
**biological_process**	GO:0060965	negative regulation of gene silencing by miRNA
