# Supplementary figures and images for: GOLink: Finding Cooccurring Terms across Gene Ontology Namespaces
Source: Int J Genomics. 2013 Dec 31;2013:594528. doi: 10.1155/2013/594528 (PMC3892482; doi:10.1155/2013/594528)

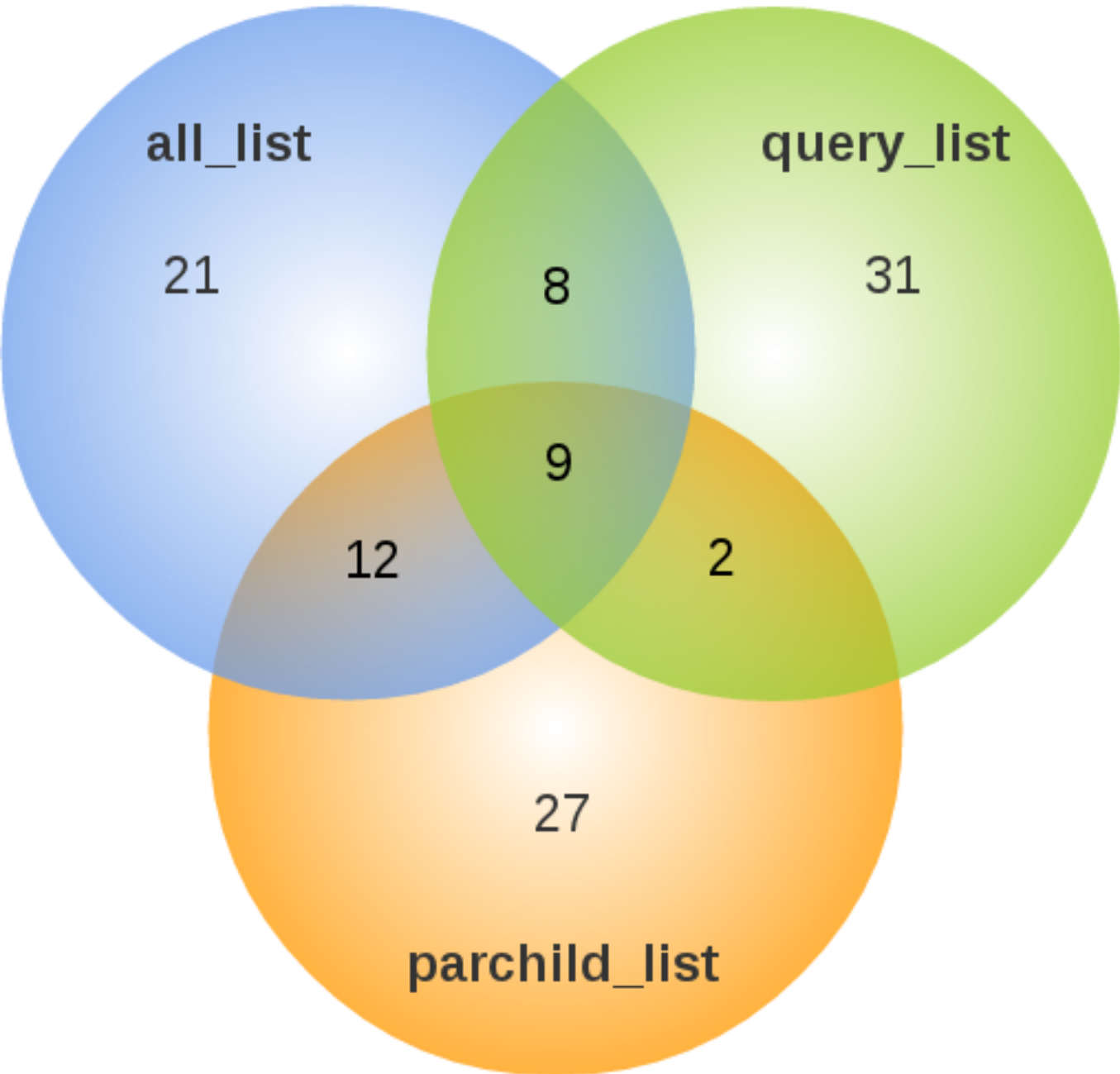

Supplement: Supplementary file 1 — (SupplementaryFile1.xls): GOLink terms lists generated using the query term ‘regulation of gene expression', the evidence code filter “!IEA” and database filter ‘UniProtKB'. Supplementary File 2 (SupplementaryFile2.xls): The top 50 Molecular Function terms from each GOLink terms list (a); the genes and annotation used for the positive list and the negative list (b); the raw statistical data for sensitivity, specificity, accuracy, PPV, NPV and the number and proportion of positive and negative genes found (c). These latter data correspond to Figure 3. Supplementary File 3 (SupplementaryFile3.xls): The genes and annotation used for the positive list and the negative list and raw statistical data for sensitivity, specificity, accuracy, PPV, NPV and the number and proportion of positive and negative genes found when the true positives from Supplementary Table 2 are removed (a). The raw statistical data when both the true positives and the term ‘protein binding' are removed from the GOLink terms lists (b). Supplementary File 4 (SupplementaryFile4.xls): The five GOLink all_list terms lists generated using the query term ‘regulation of gene expression' and the evidence code filter “!IEA” for each of the five annotation sources (“UniprotKB, “PomBase”, “MGI”, “SGD” and “ZFIN”) used in the creation of Figure 4. Supplementary File 5 (SupplementaryFile5.xls): a) Summary of the top 100 terms from a GOLink query_list terms list (b) and a QuickGO terms list (c) generated using the query term ‘regulation of gene expression' and the evidence code filter “!IEA”. Supplementary Figure 1 (SupplementaryFigure1.jpg): Venn diagram showing the overlap between the top 50 Molecular Function terms from each of the three GOLink terms lists as found in Supplementary File 2. Supplementary Table 1 (SupplementaryTable1.doc): The 59 GOLink consensus terms found in all three GOLink terms lists. Supplementary Table 2 (SupplementaryTable2.doc): Genes from the negative list deemed t [file 594528.f1.zip › Figures/SupplementaryFigure1.pdf]
